# Adaptive RGB–VNIR spectral fusion for non-destructive quality assessment of Canadian wild rice kernels

**DOI:** 10.3389/fpls.2026.1973124

**Published:** 2026-10-01

**Authors:** Zhenhua Qiao, Jinting Zou, Dongxu Wang, Chunyun Wang

**Affiliations:** The Tourism College of Changchun University, College of Artificial Intelligence, Changchun, China

**Keywords:** Canadian wild rice, lightweight network, multimodal learning, non-destructive classification, pre-storage screening, RGB–VNIR fusion

## Abstract

**Introduction:**

Canadian wild rice kernels exhibit diverse postharvest variations, including breakage, discoloration, insect damage, and incomplete dehulling, making accurate and non-destructive classification important for screening and grading. However, RGB imaging may be insufficient for distinguishing kernels with subtle or overlapping visual characteristics, while visible–near-infrared (VNIR) spectra provide complementary information but contain substantial redundancies. Moreover, conventional multimodal fusion strategies based on direct concatenation or fixed weighting cannot readily adapt to sample-specific differences in modality contribution.

**Methods:**

To address these limitations, this study proposed a lightweight RGB–VNIR dual-modal fusion framework for fine-grained quality-category classification of Canadian wild rice kernels. A Coordinate Attention-enhanced MobileNetV3-Small was employed to extract spatial phenotypic features, while a 1D-GhostResNet-SE was used to model continuous VNIR spectral responses. A Cross-Modal Contribution-Aware Recalibration Fusion Head (CMCR-FH) was further introduced to adaptively regulate the contributions of the two modalities. The framework was evaluated on 2,048 paired RGB–VNIR samples from eight categories using stratified five-fold cross-validation.

**Results:**

The proposed framework achieved a mean accuracy of 98.68% ± 0.22% and a Macro-F1 score of 98.69% ± 0.22%, representing numerical improvements of 4.93 and 4.97 percentage points, respectively, over the baseline model, while requiring only 1.236 M parameters and 63.537 M multiply–accumulate operations. Ablation experiments demonstrated complementary contributions from visual feature enhancement, lightweight spectral representation, and sample-adaptive multimodal fusion, with CMCR-FH producing the largest numerical performance gain among the evaluated components. Grad-CAM++ and SHAP analyses further indicated that the model jointly utilized visible phenotypic abnormalities and class-dependent spectral response patterns for discrimination.

**Discussion:**

These results demonstrate that integrating complementary RGB and VNIR information with sample-adaptive fusion can improve fine-grained quality-category classification while maintaining low computational complexity. The proposed framework provides an effective balance among classification accuracy, computational efficiency, and interpretability, offering a promising approach for multimodal postharvest quality-category classification and grading of Canadian wild rice kernels.

## 1 Introduction

Canadian wild rice (*Zizania palustris*) (Yang et al., 2025) is a distinctive aquatic cereal with high nutritional value and considerable commercial potential (Surendiran et al., 2014; Li et al., 2026). Kernel integrity and visual appearance are key attributes for pre-storage quality assessment and the management of storage batches (Guo et al., 2025). During harvesting, drying, dehulling, and transportation, mechanical stress, insect infestation, and quality deterioration may result in kernel breakage, insect-induced damage, and abnormal discoloration. Variations in dehulling status may further increase the compositional heterogeneity of individual batches (Gao et al., 2025). These abnormalities not only impair the visual quality and commercial value of wild rice but also complicate pre-storage screening, batch-level quality assessment, and subsequent processing. Therefore, the rapid identification of kernels exhibiting different quality conditions is essential for maintaining the uniformity of stored wild rice batches and improving the efficiency of postharvest quality management (Wang et al., 2025; Sokra et al., 2026).

Currently, the pre-storage quality inspection of Canadian wild rice relies predominantly on manual visual assessment (Eliwa and Abd El-Hafeez, 2025; Lu et al., 2025). However, the reliability of this approach is readily affected by operator experience, illumination conditions, kernel orientation, and defect conspicuity. Kernels with subtle discoloration, localized mechanical breakage, or insect-induced damage often exhibit only minor phenotypic differences, making them particularly susceptible to misclassification or missed detection during manual inspection. Consequently, it remains difficult to accurately determine the composition of kernels with different quality conditions within heterogeneous storage batches. An objective, rapid, and non-destructive detection method capable of jointly characterizing kernel appearance and spectral responses is therefore needed to support pre-storage quality screening and batch management (Yang and Tseng, 2025).

Hyperspectral imaging captures reflectance information across a continuous range of wavelengths (Song et al., 2025), providing an effective means of distinguishing samples that exhibit similar external appearances but differ in tissue properties or chemical composition (Yang and Tseng, 2025). For instance, Li et al. (2025b) combined hyperspectral imaging with deep learning to achieve the non-destructive discrimination of 500 common bean varieties, obtaining a test-set accuracy of 93.06%. Zhang et al. (2025a) employed near-infrared hyperspectral imaging to discriminate among 30 maize seed varieties, demonstrating that spectral features can provide effective discriminatory information for seeds with highly similar visual appearances. Chen et al. (2024) applied hyperspectral imaging to the quality assessment and varietal classification of pecan seeds, further demonstrating its potential for the comprehensive evaluation of seed quality attributes and varietal differences. Wang and Song (2023) combined hyperspectral imaging with deep learning to achieve varietal identification of sweet corn seeds, further demonstrating the effectiveness of data-driven spectral analysis for distinguishing seed varieties. He et al. (2019) employed near-infrared hyperspectral imaging combined with informative wavelength selection to achieve the non-destructive assessment of rice seed vigor, demonstrating the potential of characteristic spectral bands for efficient seed quality evaluation. Collectively, these studies have demonstrated the effectiveness of hyperspectral information for seed identification and quality assessment. Nevertheless, the substantial redundancy inherent in high-dimensional spectral data and the complexity of hyperspectral data processing may compromise computational efficiency and limit the generalization capability of the resulting models (Zhang et al., 2025b; Jing et al., 2026).

RGB-based machine vision provides a cost-effective means of capturing kernel color, morphological, and textural information and has therefore been widely applied to the external quality inspection of seeds and agricultural products. Islam et al. (2023) achieved varietal discrimination of tomato seeds using image-derived color and texture features, demonstrating the effectiveness of visible appearance characteristics for seed classification. Shi et al. (2026) achieved varietal identification of commercial dried goji berries by integrating image enhancement techniques with a lightweight deep learning model, demonstrating the potential of efficient machine vision approaches for the rapid classification of processed agricultural products. Mukasa et al. (2022) employed RGB imaging and deep learning to discriminate watermelon seeds with different ploidy levels, demonstrating that image-derived phenotypic features can support the rapid and non-destructive identification of seed ploidy.

Although RGB imaging offers the advantages of rapid acquisition, relatively low cost (Sreekumar et al., 2026), and convenient deployment, its discriminative information is derived primarily from visible phenotypic characteristics. When different categories exhibit only subtle color variations, localized damage, or similar textural patterns, RGB information alone may be insufficient to fully characterize the underlying quality differences (Xia et al., 2024; Guo et al., 2026). RGB images and spectral data are inherently complementary in their information representation: the former mainly capture kernel morphology, color, and surface texture, whereas the latter characterize wavelength-dependent reflectance variations across continuous spectral bands, thereby providing additional information for distinguishing samples with similar external appearances but different tissue conditions or compositional attributes. Previous studies have shown that multisource information fusion can improve the accuracy and robustness of complex agricultural product recognition tasks. Bi et al. (2025) integrated image, hyperspectral, and low-field nuclear magnetic resonance information for maize kernel variety identification; Xie et al. (2026) combined hyperspectral imaging, electronic-nose sensing, and machine vision to improve maize seed vigor classification; Li et al. (2025a) employed machine vision together with multiple optical sensing modalities to assess maize seed viability, with the fusion model outperforming the corresponding single-modality approaches; and Jeong et al. (2026) integrated near-infrared spectroscopy with machine vision for the geographical-origin authentication of agricultural products. Nevertheless, existing studies have primarily focused on variety identification, seed vigor assessment, viability detection, and geographical-origin authentication, whereas the simultaneous fine-grained recognition of multiple postharvest defect categories remains comparatively underexplored.

Canadian wild rice kernels exhibit an elongated morphology and substantial variability in surface coloration, dehulling status, and localized defect characteristics, resulting in considerable feature overlap among different quality categories. Meanwhile, paired RGB–VNIR datasets specifically developed for the pre-storage quality assessment of Canadian wild rice remain scarce. Because RGB-derived spatial representations and VNIR spectral features differ markedly in dimensionality, feature scale, and category-specific discriminative contribution, conventional fusion strategies based on direct concatenation or fixed weighting may fail to fully exploit the complementary information provided by the two modalities. Canadian wild rice kernels exhibit an elongated morphology and substantial variability in surface coloration, dehulling status, and localized defect characteristics, resulting in considerable feature overlap among different quality categories. Meanwhile, paired RGB–VNIR datasets specifically developed for the pre-storage quality assessment of Canadian wild rice remain scarce. Because RGB-derived spatial representations and VNIR spectral features differ markedly in dimensionality, feature scale, and category-specific discriminative contribution, conventional fusion strategies based on direct concatenation or fixed weighting may fail to fully exploit the complementary information provided by the two modalities.

To address these challenges, this study employed a publicly available paired RGB–VNIR multimodal dataset of Canadian wild rice kernels and developed a dual-modality quality recognition model. The proposed model adaptively integrates appearance-related spatial representations with wavelength-dependent spectral features, enabling the non-destructive discrimination of normal kernels and multiple defect categories. The resulting framework provides a methodological basis for investigating RGB–VNIR-based pre-storage quality classification of wild rice kernels.

## 2 Materials and methods

### 2.1 Sample introduction

This study employed Canadian wild rice (*Zizania palustris*) kernels from a publicly available RGB–VNIR multimodal dataset. The original samples were collected by the dataset developers from four Canadian provinces—Manitoba, Ontario, Saskatchewan, and Alberta—and consisted of postharvest wild rice kernels intended for commercial processing and quality grading. Prior to imaging, the samples were manually cleaned to remove debris and foreign materials without washing. The cleaned kernels were stored at 4 °C and imaged at room temperature to preserve their natural postharvest condition. Based on kernel size, degree of surface discoloration, severity of structural damage, signs of insect infestation, and hull-retention status, the samples were classified into eight quality categories. Representative images of the corresponding categories are shown in **Figure 1**, and the detailed classification criteria are presented in **Table 1**. These categories represent the common size variations, visual abnormalities, and structural defects encountered in the postharvest commercial grading of wild rice (Sikiru and Erkinbaev, 2026).

**Figure 1:**
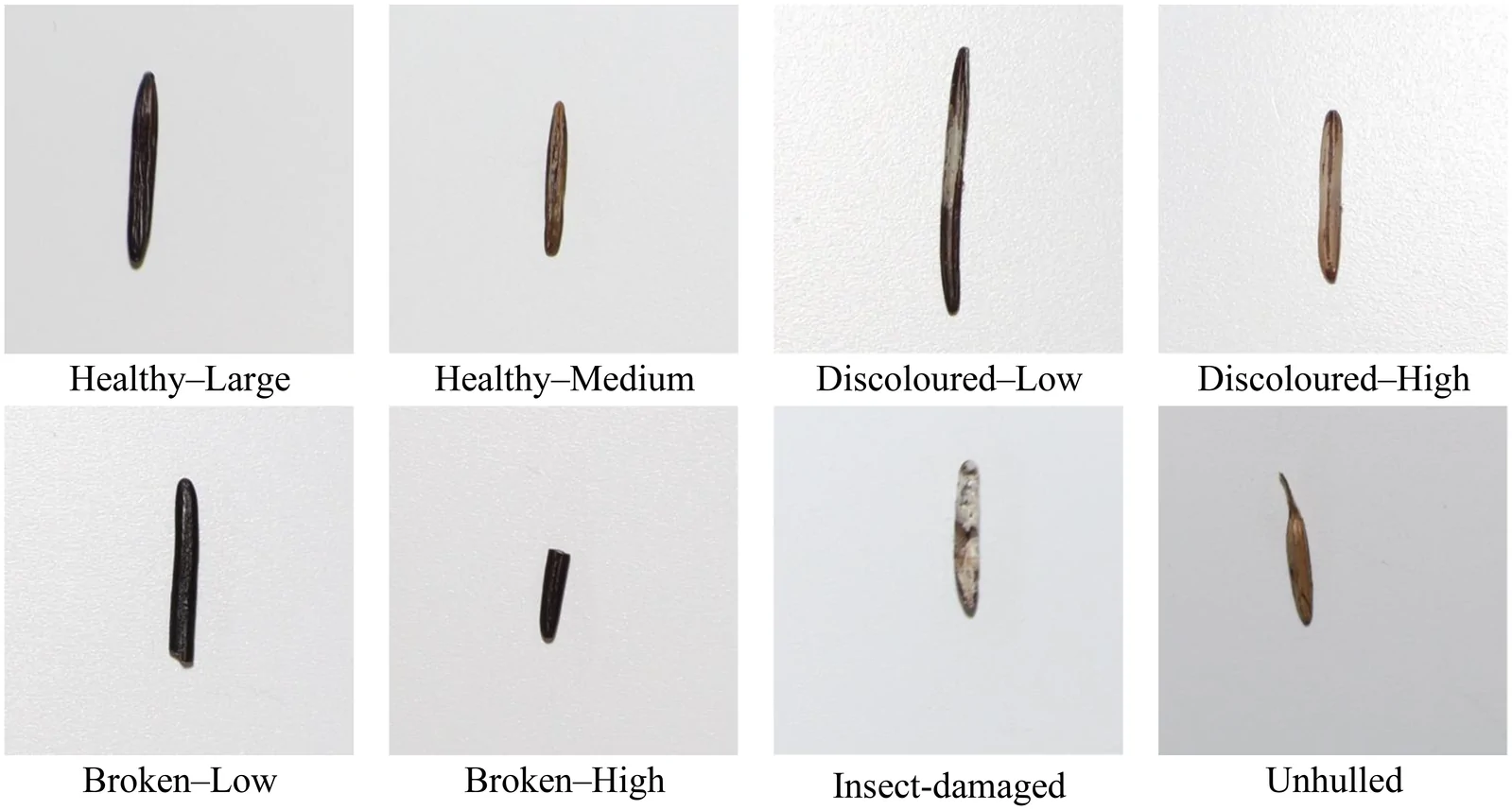
Representative RGB images of the eight Canadian wild rice kernel quality categories.

**Table 1:** Quality categories and classification criteria of Canadian wild rice kernels.

Class label	Classification criterion
Healthy–Large	Healthy kernels typically measuring approximately 18–20 mm or more in length and 2.0–2.2 mm or more in width.
Healthy–Medium	Healthy kernels typically measuring approximately 14–16 mm in length and 1.6–2.0 mm in width.
Discoloured–Low	Kernels exhibiting mild, localized discoloration affecting less than approximately 30% of the visible surface.
Discoloured–High	Kernels exhibiting extensive or pronounced discoloration over a substantial proportion of the visible surface.
Broken–Low	Kernels with minor cracks or edge chipping while largely retaining their original morphology.
Broken–High	Kernels with severe structural damage, including major splits extending over more than 50% of the kernel length.
Insect-damaged	Kernels showing visible signs of insect activity, such as perforations, feeding marks, or localized tissue loss.
Unhulled	Kernels retaining the outer hull because of incomplete dehulling during processing.

### 2.2 Data collection and preprocessing

For the paired subset used in this study, each RGB image was matched one-to-one with its corresponding VNIR hyperspectral sample using a unique kernel identifier, ensuring that both modalities represented the same kernel. The overall workflow of sample preparation, RGB image acquisition, VNIR hyperspectral acquisition, and paired data generation is illustrated in **Figure 2**.

**Figure 2:**
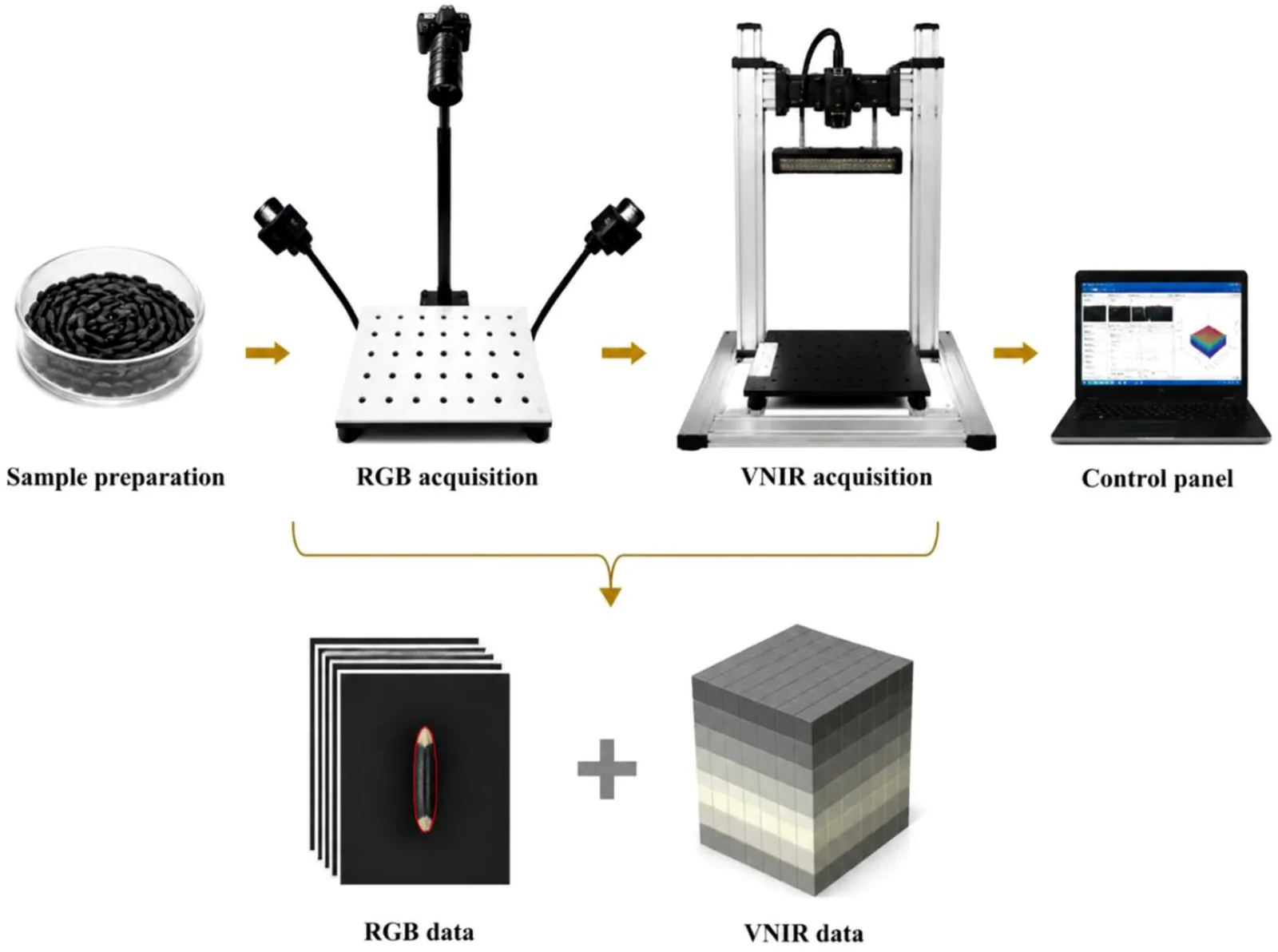
RGB–VNIR data acquisition workflow for Canadian wild rice.

RGB images were acquired using a Canon EOS R100 mirrorless camera equipped with an 18–45 mm STM lens. The camera was mounted in a fixed top-down configuration, and all images were captured using consistent settings: a spatial resolution of 4000 × 4000 pixels, an aperture of f/5.6, an exposure time of 8 ms, and a focal length of 30 mm. The kernels were arranged in a 6 × 6 grid on a white background, with each kernel placed in an individual cell to prevent overlap. Two halogen lamps were positioned symmetrically at approximately 45° to the sample plane to provide uniform illumination. After acquisition, individual kernels were cropped from the full-frame images and resized to 512 × 512 pixels.

VNIR hyperspectral data were acquired using a Specim FX pushbroom imaging system operating over the 400–1000 nm spectral range. The system recorded 224 contiguous spectral bands with a sampling interval of approximately 2.7 nm and a spectral resolution of 5.5 nm. The hyperspectral images had a spatial dimension of 387 × 512 pixels and a radiometric resolution of 12 bit. The integration time and acquisition rate were set to 25 ms and 20 fps, respectively, with 2 × 2 spatial binning. During acquisition, the kernels were scanned continuously on a conveyor belt under halogen illumination.

For each hyperspectral scan, dark and white reference measurements were collected to correct for sensor dark current, illumination non-uniformity, and system-response variations. The dark reference was acquired with the camera shutter closed, whereas the white reference was obtained using a 99% Spectralon reflectance standard. According to the original dataset documentation, the hyperspectral data were converted to reflectance using dark–white reference calibration, and single-kernel spectra were subsequently extracted from the calibrated hyperspectral data. In the present study, the provided single-kernel VNIR spectra were used directly for subsequent preprocessing and modeling. To improve spectral stability, three bands with relatively high noise at the spectral boundaries were excluded before subsequent processing, leaving 221 bands for VNIR feature extraction. No additional normalization or standardization was applied to the retained VNIR spectra before model input.

The dataset comprised 2,048 paired RGB–VNIR samples from eight quality categories, with 256 samples per category. Model generalization was evaluated using a fixed five-fold cross-validation scheme with acquisition-image-level grouping. Specifically, kernels originating from the same original acquisition image were treated as a single group and were assigned collectively to the same fold. Thus, kernels derived from the same acquisition image were never distributed between the training and validation sets within a given cross-validation round. The groups were allocated across five mutually exclusive folds while maintaining the class distribution as closely as possible. In each round, one-fold was used for validation and the remaining four folds for training. The detailed fold assignments and corresponding numbers of training and validation samples are presented in **Table 2**. Each RGB image and its corresponding VNIR spectrum for an individual kernel were treated as an inseparable pair and were always assigned to the same fold. Final performance was reported as the mean ± standard deviation of Accuracy, Macro-Precision, Macro-Recall, and Macro-F1 across the five validation folds.

**Table 2:** Data partitioning for stratified five-fold cross-validation.

Fold	Training folds	Validation fold	Number of training samples	Number of validation samples
Fold 1	F2–F5	F1	1,638	410
Fold 2	F1、F3–F5	F2	1,638	410
Fold 3	F1–F2、F4–F5	F3	1,638	410
Fold 4	F1–F3、F5	F4	1,639	409
Fold 5	F1–F4	F5	1,639	409

### 2.3 Average reflectivity

The figure shows the mean visible–near-infrared reflectance spectra of eight Canadian wild rice quality categories over the measured wavelength range of 400.28–998.17 nm. Each curve represents the wavelength-wise mean reflectance of all kernels within the corresponding category. Mean reflectance generally increased with wavelength, with pronounced changes occurring between approximately 680 and 750 nm. Unhulled kernels exhibited the highest mean reflectance beyond approximately 550 nm, followed by Discoloured–High kernels. Insect-damaged and Discoloured–Low kernels showed intermediate reflectance responses. Healthy–Large kernels had the lowest overall mean reflectance. Healthy–Medium, Broken–Low, and Broken–High kernels exhibited relatively similar spectral profiles but remained distinguishable in the near-infrared region. As shown in **Figure 3**.

**Figure 3:**
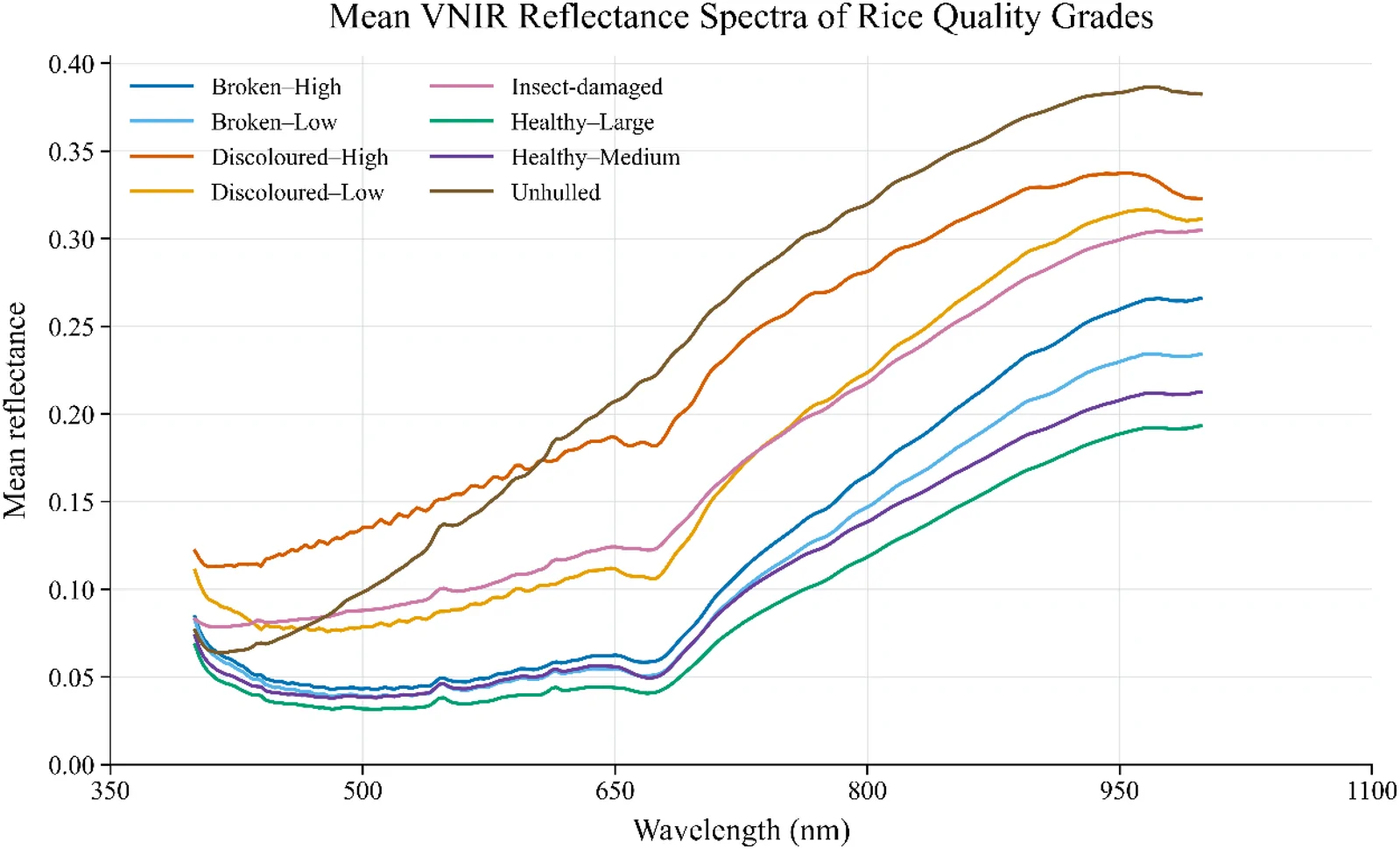
Mean VNIR reflectance spectra of different Canadian wild rice quality categories.

### 2.4 Comparative experimental design

#### 2.4.1 Overall structure

This study proposes a lightweight RGB–VNIR dual-modal fusion framework for the quality classification of wild rice kernels. As shown in **Figure 4** By jointly modeling visual–spatial information and spectral response characteristics, the proposed framework addresses the limited representation capability of individual modalities in fine-grained quality recognition. The overall architecture consists of an enhanced visual encoding branch, a lightweight spectral encoding branch, and a cross-modal contribution-aware fusion module. For RGB modality, MobileNetV3-Small is employed as the visual feature extraction backbone. A Coordinate Attention (CA) module is incorporated at the end of the backbone to enhance spatial position awareness and improve the selection of informative regions. While maintaining a lightweight architecture and high computational efficiency, the RGB branch can extract discriminative visual representations. A projection head is subsequently used to map the visual features into a shared embedding space, thereby facilitating cross-modal feature interaction. For the VNIR modality, a lightweight spectral encoding branch is designed by integrating Ghost Conv1D, one-dimensional Squeeze-and-Excitation attention, and residual connections. This branch effectively captures discriminative wavelength patterns from continuous spectral responses and strengthens the representation of informative spectral channels. By reducing computational complexity while preserving wavelength-dependent information, the spectral branch produces compact and discriminative VNIR representations. During multimodal fusion, a Cross-Modal Contribution-Aware Recalibration Fusion Head (CMCR-FH) is introduced to adaptively estimate the contribution of the RGB and VNIR modalities for each input sample. The module dynamically learns modality-specific contribution weights, recalibrates the corresponding feature representations, and integrates complementary visual and spectral information. Compared with conventional feature concatenation, CMCR-FH suppresses interference from redundant information and improves the discriminative capability of the joint spatial–spectral representation.

**Figure 4:**
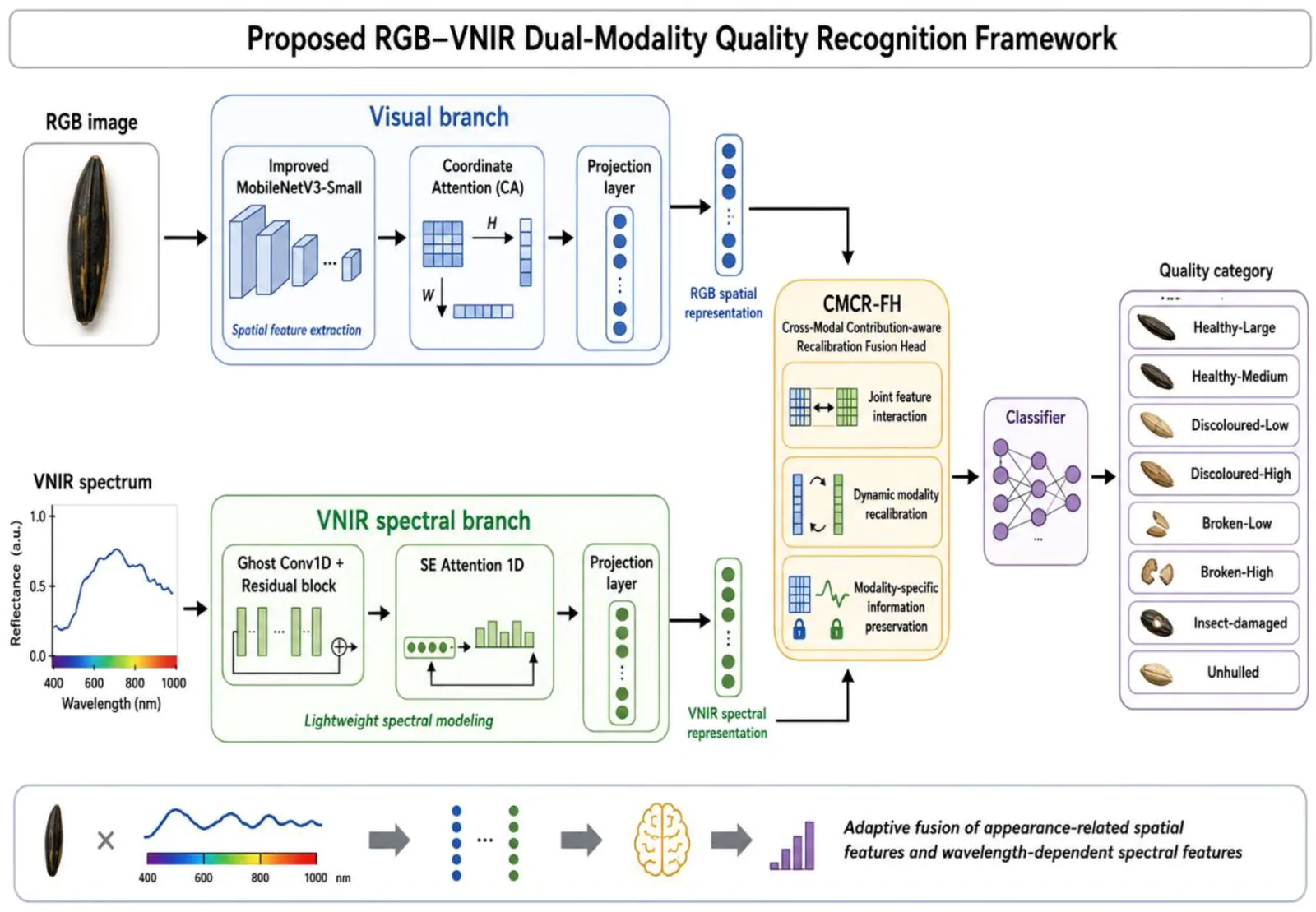
Overall architecture of the proposed RGB–VNIR dual-modality quality recognition model.

Finally, the fused representation is fed into a classifier to perform eight-class wild rice kernel quality classification. In addition, Grad-CAM visualization, spectral importance analysis, and modality contribution weight visualization are employed to interpret the model’s decision-making process.

#### 2.4.2 Coordinate attention-enhanced MobileNetV3-small

To address the limited spatial representation capability of lightweight convolutional networks in complex visual scenarios, this study develops a Coordinate Attention-enhancedMobileNetV3-Small(CA-MobV3S) (Hou et al., 2021) feature extraction module. MobileNetV3-Small employs depthwise separable convolutions and inverted residual bottleneck structures to achieve efficient feature extraction with reduced computational complexity. However, its original Squeeze-and-Excitation (SE) attention mechanism mainly models channel-wise dependencies through global feature compression, which may weaken spatial positional information and limit the network’s ability to capture discriminative local regions (Howard et al., 2019).

To overcome this limitation, a Coordinate Attention (CA) module is incorporated into the final feature extraction stage of MobileNetV3-Small, between the last convolutional feature map and the global average pooling layer. Unlike conventional channel attention mechanisms that only estimate the importance of different channels, CA decomposes spatial encoding into two directional components and captures long-range dependencies along horizontal and vertical directions. This design enables the network to establish a joint representation of channel responses and spatial positional information. As shown in **Figure 5**.

**Figure 5:**
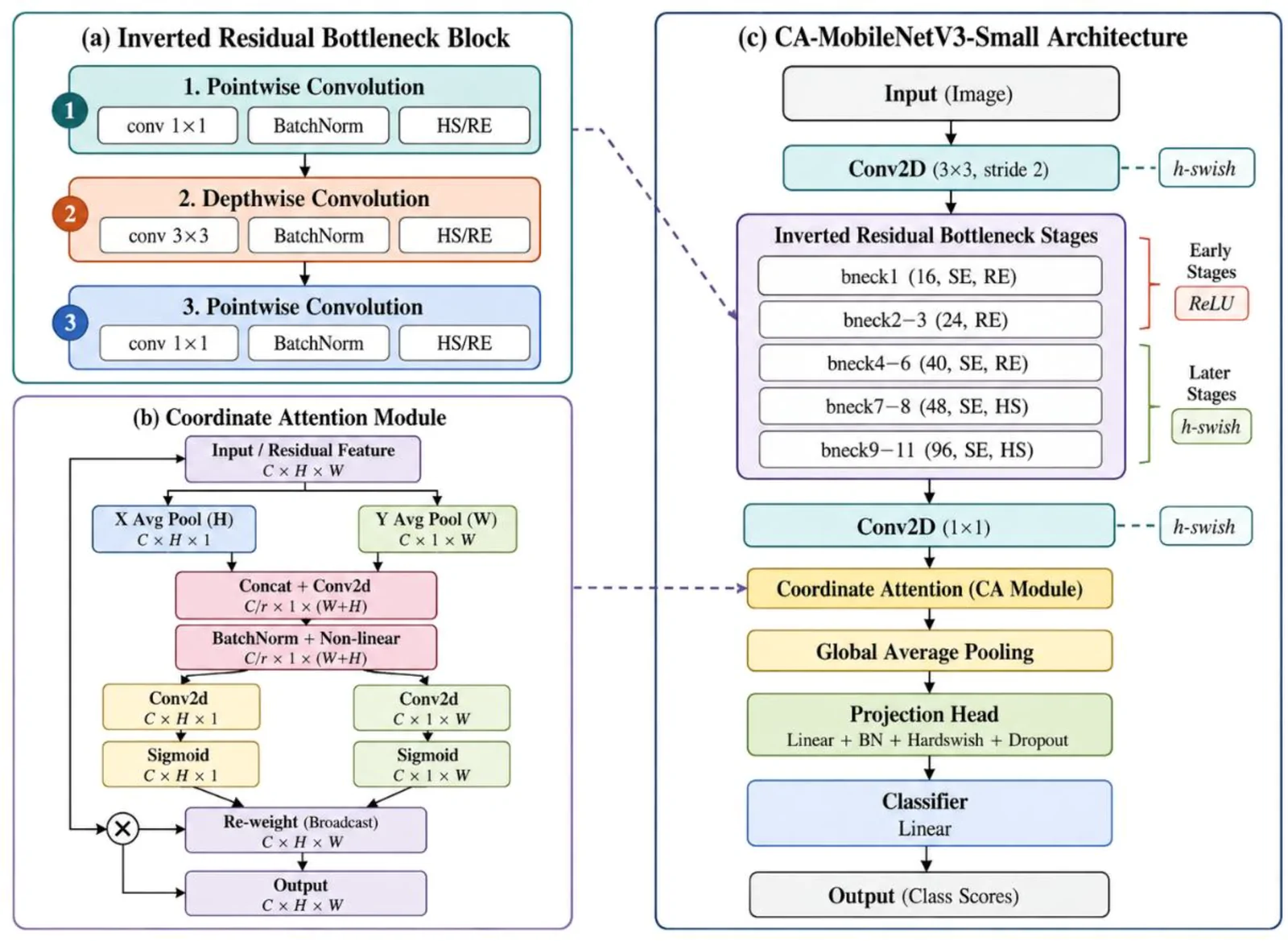
Architecture of the CA-MobileNetV3-Small visual branch and its key modules: **(a)** inverted residual bottleneck block; **(b)** coordinate attention module; and **(c)** CA-MobileNetV3-Small architecture.

Given the feature map extracted by MobileNetV3-Small, the feature representation is defined in Equation 1:

(1) $$\text{X}\in {\text{R}}^{\text{C}\times \text{H}\times \text{W}}$$

The CA module generates direction-aware attention weights along the height and width dimensions, and the enhanced feature representation is obtained according to Equation 2:

(2) $$Y=X⊙g_{h}⊙g_{w}$$

where $g_{h}$and $g_{w}$denote the attention weights generated along the height and width directions, respectively, and ⊙ represents element-wise multiplication. Through this coordinate-aware feature recalibration process, informative regions are strengthened while less relevant responses are suppressed.

Compared with increasing network depth or introducing additional convolutional layers, the proposed CA enhancement provides an efficient feature refinement strategy with limited computational overhead. The improved MobileNetV3-Small maintains its lightweight characteristics while achieving stronger spatial perception and more discriminative feature representation, making it more suitable for fine-grained visual recognition tasks.

#### 2.4.3 1D-GhostResNet-SE

VNIR spectra contain strong correlations between adjacent wavelengths and substantial redundant information. To obtain compact yet discriminative spectral representations, a lightweight 1D-GhostResNet encoder is constructed by integrating Ghost Conv1D, SE Attention 1D, and residual learning. Given an input feature $\text{X}\in {\mathbb{R}}^{{\text{C}}_{\text{in}}\times \text{L}}$, the main pathway employs two successive Ghost Conv1D layers to capture local spectral dependencies with reduced computational redundancy. The resulting features are subsequently processed by SE Attention 1D, which adaptively emphasizes informative spectral feature channels while suppressing less relevant responses (Targ et al., 2016; Han et al., 2020; Guo et al., 2022).

A shortcut pathway is introduced to preserve the original spectral information and facilitate gradient propagation. The output of each residual block is expressed in Equation 3.

(3) $$Y=\text{ReLU}(F_{\text{Ghost-SE}}(X)+F_{\text{shortcut}}(X))$$

where $F_{\text{Ghost-SE}}(X)$denotes the transformation perfrmed by two successive Ghost Conv1D layers followed by SE Attention 1D, and $F_{\text{shortcut}}(X)$represents the residual mapping. When the input and output dimensions are identical, the shortcut is implemented as an identity mapping; otherwise, a 1×1Conv1D is used for dimensional alignment. By combining efficient feature generation, adaptive channel recalibration, and residual information preservation, the proposed encoder produces a lightweight and discriminative VNIR representation for subsequent multimodal fusion. As shown in **Figure 6**.

**Figure 6:**
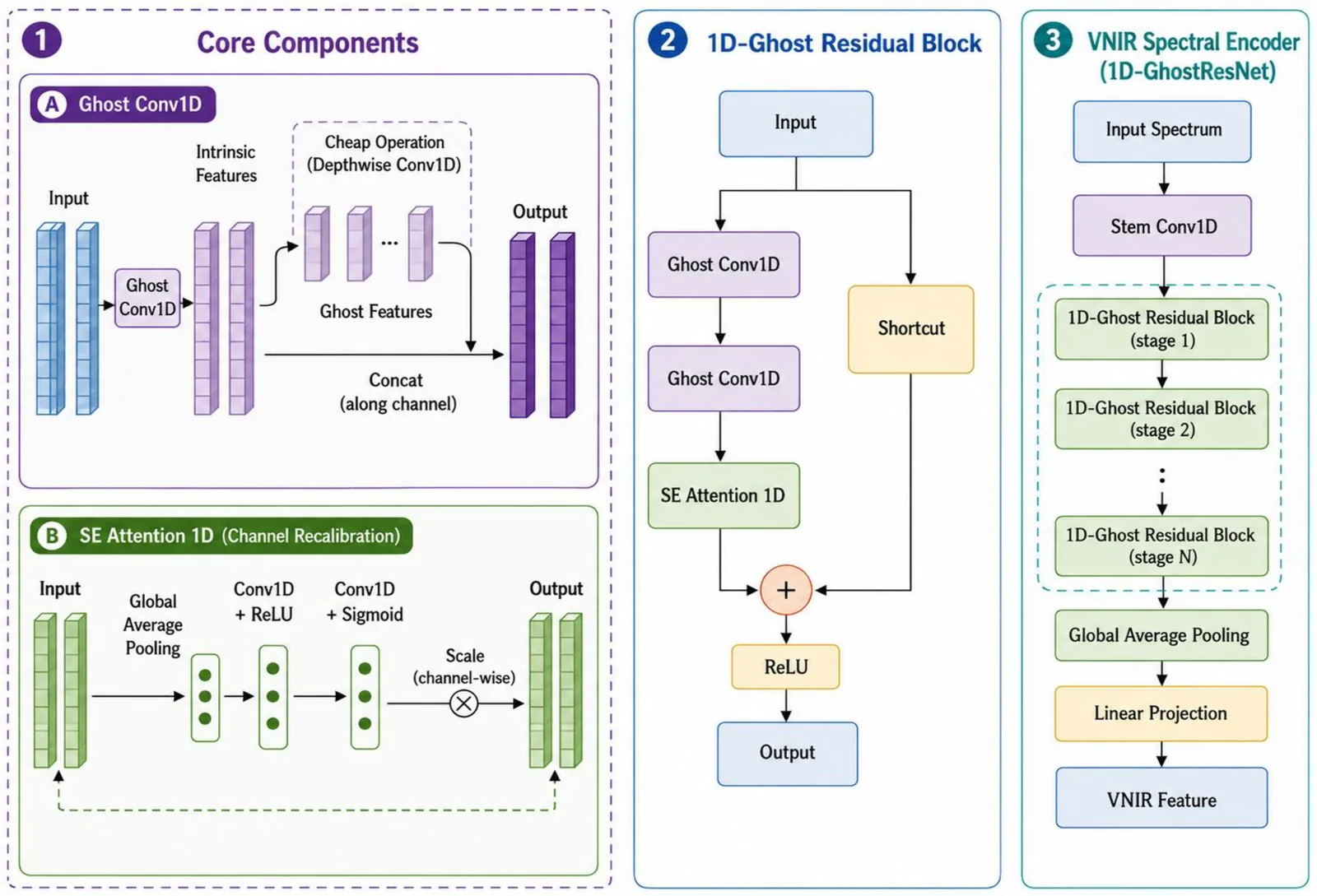
Architecture of the VNIR spectral encoder and its core components: (1) core components, including **(A)** Ghost Conv1D and **(B)** SE Attention 1D; (2) 1D-Ghost residual block; and (3) VNIR spectral encoder (1D-GhostResNet).

#### 2.4.4 Cross-modal contribution-aware recalibration fusion head

To adaptively integrate complementary information from RGB and VNIR modalities, we design a Cross-Modal Contribution-Aware Recalibration Fusion Head (CMCR-FH) as shown in **Figure 7**. Given the modality-specific features ${\text{F}}_{r},{\text{F}}_{v}\in {\mathbb{R}}^{B\times D}$, they are first concatenated to form a joint cross-modal representation. A lightweight contribution estimation branch, implemented using two fully connected layers with ReLU activation and dropout regularization, predicts two normalized modality contribution coefficients according to Equation 4:

**Figure 7:**
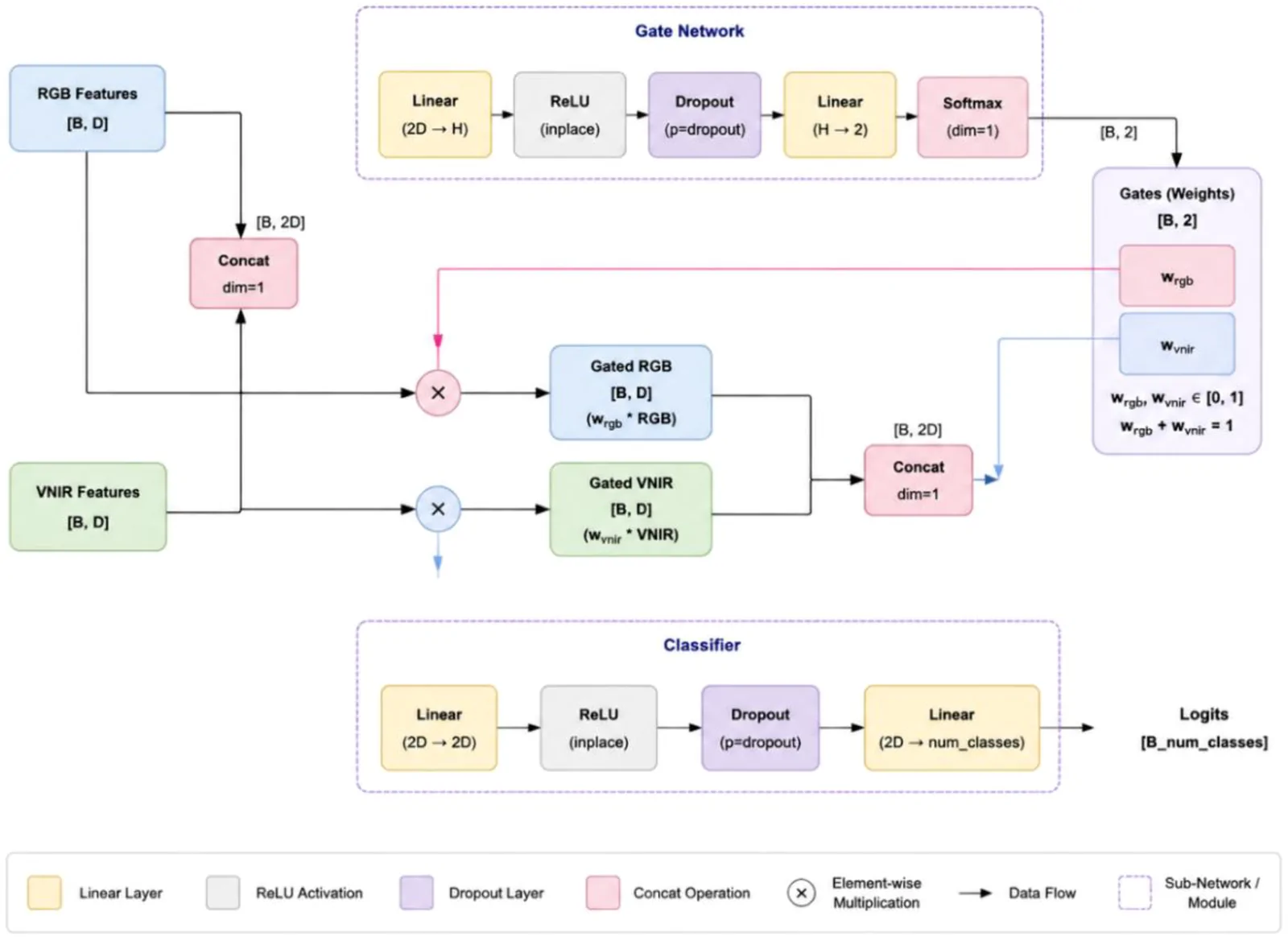
Architecture of the proposed CMCR-FH adaptive dual-modality fusion module.

(4) $$\left[\alpha_{r},\alpha_{v}]=\text{Softmax}({\text{FC}}_{2}(\text{Dropout}(\text{ReLU}({\text{FC}}_{1}([{\text{F}}_{r}∥{\text{F}}_{v}]))))\right)$$

where $\alpha_{r}+\alpha_{v}=1$. The two coefficients are estimated independently for each sample and represent the relative contributions of the RGB and VNIR modalities. They are scalar modality-level coefficients shared across all feature dimensions.

The original features are then recalibrated using their corresponding coefficients and concatenated to construct the fused representation, as formulated in Equation 5:

(5) $${\text{F}}_{f}=[\alpha_{r}⊙{\text{F}}_{r}∥\alpha_{v}⊙{\text{F}}_{v}]$$

where ⊙denotes broadcast multiplication and ∥denotes concatenation. Unlike weighted-sum fusion, this operation preserves the modality-specific representations after recalibration. Finally, ${\text{F}}_{\text{f}}$is processed by a nonlinear classifier composed of a fully connected layer, ReLU activation, dropout, and an output layer. Consequently, CMCR-FH dynamically adjusts the relative influence of the two modalities for each sample while retaining their complementary information for final prediction.

### 2.5 Evaluation metrics

This research employs multiple metrics to evaluate model performance, including Accuracy, Precision, Recall, F1 score, Parameters, MACs, and the Confusion Matrix. For a dataset D with n samples, these metrics are calculated as follows:

Accuracy is calculated according to Equation 6:

(6) $$\text{Accuracy}=\frac{\sum_{i=1}^{n}I(y_{i}={\hat{y}}_{i})}{n}$$

where ${\text{y}}_{\text{i}}$represents the true label of the i-th sample, ${\hat{\text{y}}}_{\text{i}}$denotes the predicted label of the i-th sample, and I(·)is an indicator function that returns 1 when ${\text{y}}_{\text{i}}={\hat{\text{y}}}_{\text{i}}$and 0 otherwise.

Precision is calculated according to Equation 7:

(7) $$\text{Precision}=\frac{TP}{TP+FP}$$

Recall is calculated according to Equation 8:

(8) $$\text{Recall}=\frac{TP}{TP+FN}$$

where TP(True Positive) represents the number of correctly predicted positive samples, FP(False Positive) represents the number of incorrectly predicted positive samples, and FN(False Negative) represents the number of incorrectly predicted negative samples.

The F1 score is calculated according to Equation 9:

(9) $$F1=\frac{2\times \text{Precision}\times \text{Recall}}{\text{Precision}+\text{Recall}}$$

### 2.6 Implementation details and pretraining strategy

A fixed stratified five-fold cross-validation scheme was adopted, with all candidate models trained independently under identical settings except for the factor under investigation. For initialization of the RGB branch, task-specific pretraining was performed using the RGB-only portion of the original dataset that was independent of the paired RGB–VNIR subset used for multimodal model development and evaluation. The complete RGB dataset contained 16,000 original single-kernel images. Before RGB pretraining, all 2,048 kernels included in the paired RGB–VNIR dataset were identified according to their unique kernel identifiers and excluded from the pretraining pool. Consequently, the RGB pretraining set consisted of the remaining 13,952 single-kernel RGB images. None of the 2,048 paired samples subsequently used in the five-fold cross-validation, including the samples assigned to each validation fold, was involved in RGB pretraining. The same predefined cross-validation partitions were used throughout the controlled model-comparison and ablation experiments to ensure consistent evaluation conditions across candidate configurations. Because these cross-validation results were also used to guide model-component selection, the reported metrics are interpreted as cross-validated validation performance within the present development dataset rather than as performance obtained from an independent test set. The principal training configurations are summarized in **Table 3**, and all experiments were conducted using Python 3.8.20, PyTorch 2.2.2, and CUDA 11.8 on an NVIDIA GeForce GTX 1080 Ti GPU.

**Table 3:** Main training configurations for the complete model and controlled experiments.

Item	Setting
RGB input size	224 × 224 pixels
VNIR input dimensionality	221
Number of classes	8
RGB branch initialization	Pretrained on 13,952 non-paired RGB kernels
RGB branch fine-tuning strategy	Frozen for the first 5 epochs, followed by unfreezing and joint fine-tuning
Default fusion feature dimension	128
Batch size	16
Optimizer	AdamW
Initial learning rate	1.2 × 10^-4^
Weight decay	1.5 × 10^-4^
Learning-rate schedule	Cosine annealing
Number of training epochs	80
Label smoothing	0.02
Random seed	42
Validation scheme	Stratified five-fold cross-validation

### 2.7 Model interpretability analysis

SHAP GradientExplainer was used to quantify the contribution of individual VNIR wavelengths to the multimodal predictions. The analysis included all 312 samples in the held-out test subset, comprising 39 samples from each quality category. For each target sample, its paired RGB image was held fixed, and SHAP values were calculated only with respect to variations in the normalized VNIR spectrum. Therefore, the resulting values characterize the conditional contribution of the VNIR modality given the corresponding visual input.

A class-balanced background set containing 64 normalized spectra was selected exclusively from the training subset, with eight spectra from each category. One hundred gradient samples were used for each explanation. For the global wavelength-importance curve, the absolute SHAP values were first averaged across the eight model outputs for each sample and were then averaged across all explained samples. The 95% confidence interval at each wavelength was estimated using a nonparametric percentile-bootstrap procedure. Specifically, the 312 explained samples were resampled with replacement 500 times using the original sample size in each repetition. The 2.5th and 97.5th percentiles of the resulting mean-importance distributions were used as the lower and upper confidence limits, respectively. All SHAP analyses used a fixed random seed of 42.

## 3 Experiments and analysis of results

### 3.1 Comparison experience

Controlled experiments were conducted to determine the RGB backbone, VNIR encoder, and multimodal fusion strategy of the final model. Within each experimental group, the data partitions, optimization settings, and evaluation protocol were kept unchanged, with only the component under investigation varied. The corresponding cross-validation performance, 95% confidence intervals, and computational complexity are summarized in **Table 4**.

**Table 4:** Cross-validation performance and computational complexity of the evaluated RGB backbones, VNIR spectral encoders, and multimodal fusion methods.

Experimental group	Model/method	Params (M)	MACs (M)	Accuracy (%) mean ± SD [95% CI]	Precision (%) mean ± SD [95% CI]	Recall (%) mean ± SD [95% CI]	Macro-F1 (%) mean ± SD [95% CI]
RGB backbone	MobileNetV3-Small	1.650	55.648	88.77 ± 0.67 [87.94, 89.60]	88.86 ± 0.71 [87.98, 89.74]	88.77 ± 0.66 [87.95, 89.59]	88.77 ± 0.67 [87.94, 89.60]
RGB backbone	Improved MobileNetV3-Small	1.034	61.231	90.82 ± 0.33 [90.41, 91.23]	90.87 ± 0.34 [90.45, 91.29]	90.82 ± 0.33 [90.41, 91.23]	90.81 ± 0.34 [90.39, 91.23]
RGB backbone	RepViT-M1.0	6.462	1141.969	88.67 ± 0.57 [87.96, 89.38]	88.73 ± 0.55 [88.05, 89.41]	88.67 ± 0.56 [87.97, 89.37]	88.66 ± 0.56 [87.96, 89.36]
RGB backbone	EfficientFormerV2-S0	3.270	386.459	87.45 ± 0.84 [86.41, 88.49]	87.49 ± 0.83 [86.46, 88.52]	87.46 ± 0.84 [86.42, 88.50]	87.42 ± 0.85 [86.36, 88.48]
RGB backbone	FasterNet-T0	2.790	339.530	86.57 ± 0.96 [85.38, 87.76]	86.67 ± 1.05 [85.37, 87.97]	86.58 ± 0.97 [85.38, 87.78]	86.54 ± 0.99 [85.31, 87.77]
RGB backbone	ResNet18	11.243	1823.589	88.87 ± 0.47 [88.29, 89.45]	88.93 ± 0.46 [88.36, 89.50]	88.87 ± 0.48 [88.27, 89.47]	88.84 ± 0.49 [88.23, 89.45]
VNIR encoder	MLP	0.107	0.107	85.55 ± 0.94 [84.38, 86.72]	85.68 ± 0.87 [84.60, 86.76]	85.56 ± 0.93 [84.41, 86.71]	85.54 ± 0.92 [84.40, 86.68]
VNIR encoder	1D-CNN	0.053	2.623	90.48 ± 0.55 [89.80, 91.16]	90.64 ± 0.57 [89.93, 91.35]	90.47 ± 0.54 [89.80, 91.14]	90.45 ± 0.53 [89.79, 91.11]
VNIR encoder	1D-GhostResNet-SE	0.128	4.298	91.80 ± 0.27 [91.46, 92.14]	91.85 ± 0.28 [91.50, 92.20]	91.79 ± 0.28 [91.44, 92.14]	91.78 ± 0.27 [91.44, 92.12]
VNIR encoder	1D-Transformer	0.444	124.460	88.48 ± 0.86 [87.41, 89.55]	88.68 ± 0.88 [87.59, 89.77]	88.48 ± 0.85 [87.42, 89.54]	88.46 ± 0.82 [87.44, 89.48]
VNIR encoder	PatchTST	0.618	15.930	87.84 ± 0.72 [86.95, 88.73]	87.94 ± 0.69 [87.08, 88.80]	87.84 ± 0.71 [86.96, 88.72]	87.81 ± 0.71 [86.93, 88.69]
VNIR encoder	TimesNet	0.159	33.389	84.23 ± 1.01 [82.98, 85.48]	84.34 ± 0.99 [83.11, 85.57]	84.24 ± 1.02 [82.97, 85.51]	84.22 ± 1.00 [82.98, 85.46]
Fusion method	Concat Fusion	1.203	63.504	96.04 ± 0.58 [95.32, 96.76]	96.07 ± 0.60 [95.33, 96.81]	96.04 ± 0.59 [95.31, 96.77]	96.04 ± 0.60 [95.30, 96.78]
Fusion method	Static Weighted Sum Fusion	1.153	63.454	97.61 ± 0.40 [97.11, 98.11]	97.66 ± 0.38 [97.19, 98.13]	97.61 ± 0.40 [97.11, 98.11]	97.62 ± 0.39 [97.14, 98.10]
Fusion method	Hadamard Product Fusion	1.153	63.454	95.70 ± 0.64 [94.91, 96.49]	95.78 ± 0.62 [95.01, 96.55]	95.70 ± 0.64 [94.91, 96.49]	95.72 ± 0.63 [94.94, 96.50]
Fusion method	CMCR-FH	1.236	63.537	98.68 ± 0.22 [98.41, 98.95]	98.74 ± 0.20 [98.49, 98.99]	98.68 ± 0.22 [98.41, 98.95]	98.69 ± 0.22 [98.42, 98.96]

Values are reported as mean ± SD across five folds, with 95% confidence intervals shown in brackets.

For the RGB branch, the improved MobileNetV3-Small achieved the highest mean Accuracy and Macro-F1 while reducing the parameter count from 1.650 M to 1.034 M compared with the original backbone. This result indicates that the compact projection structure and Coordinate Attention improved feature representation without substantially increasing model complexity. For VNIR spectral modeling, 1D-GhostResNet-SE yielded the highest mean Accuracy and Macro-F1, together with the smallest cross-fold variation. Its performance suggests that the combination of residual learning, Ghost feature generation, and channel-wise recalibration is effective for extracting discriminative information from one-dimensional spectral data.

With both modality-specific encoders fixed, CMCR-FH achieved an Accuracy of 98.68% ± 0.22% and a Macro-F1 of 98.69% ± 0.22%, which were numerically higher than those of Static Weighted Sum Fusion by 1.07 percentage points for both metrics. The improvement was obtained with only a marginal increase in parameters and MACs, indicating that the performance gain was mainly attributable to adaptive modality contribution recalibration rather than increased model capacity. According to the comparative results, the improved MobileNetV3-Small, 1D-GhostResNet-SE, and CMCR-FH were employed as the RGB encoder, VNIR encoder, and fusion module of the complete model, respectively.

### 3.2 Key component ablation experiment

To evaluate the contributions of Ghost-SE, CA-MobV3S, and CMCR-FH, eight configurations (A0–A7) were constructed using identical data partitions, training settings, and model-selection criteria. A0 represents the baseline without the three proposed components, whereas A7 represents the complete model. When a component was disabled, it was replaced by the corresponding standard residual block, identity mapping, or concatenation-based fusion. The results are summarized in **Table 5**.

**Table 5:** Ablation results of the key components.

Settings	Enable component	Accuracy (%)	Precision (%)	ReCA-MobV3Sll (%)	Macro-F1 (%)	Params (M)	MACs (M)
A0	NO-Ghost-SE/CA-MOBV3S/CMCR-FH	93.75 ± 0.66	93.95 ± 0.63	93.75 ± 0.66	93.72 ± 0.69	1.249	64.857
A1	Ghost-SE	95.07 ± 0.50	95.12 ± 0.48	95.07 ± 0.51	95.07 ± 0.51	1.179	63.221
A2	CA-MOBV3S	94.73 ± 0.72	94.76 ± 0.74	94.73 ± 0.73	94.70 ± 0.74	1.281	65.148
A3	CMCR-FH	95.70 ± 0.44	95.86 ± 0.45	95.71 ± 0.44	95.72 ± 0.45	1.274	64.881
A4	Ghost -SE+ CA-MOBV3S	96.04 ± 0.58	96.07 ± 0.60	96.04 ± 0.59	96.04 ± 0.60	1.212	63.513
A5	Ghost -SE+ CMCR-FH	97.36 ± 0.32	97.38 ± 0.31	97.36 ± 0.32	97.36 ± 0.33	1.204	63.246
A6	CA-MOBV3S + CMCR-FH	97.02 ± 0.47	97.12 ± 0.44	97.03 ± 0.47	97.02 ± 0.47	1.306	65.173
A7	Ghost -SE+ CA-MOBV3S + CMCR-FH	98.68 ± 0.22	98.74 ± 0.20	98.68 ± 0.22	98.69 ± 0.22	1.236	63.537

The complete model A7 achieved the highest numerical Accuracy and Macro-F1 values of 98.68% ± 0.22% and 98.69% ± 0.22%, respectively, corresponding to improvements of 4.93 and 4.97 percentage points over A0. All three components were associated with improved numerical performance under the five-fold cross-validation setting. Among them, CMCR-FH produced the largest individual numerical gain and the greatest performance decrease when removed from the complete model. In addition, A7 exhibited the lowest cross-fold variation and slightly lower computational complexity than A0. These results indicate the complementary contributions of the three components without increasing model scale. The observed differences represent numerical performance improvements and should not be interpreted as evidence of statistical significance; formal statistical comparisons require further verification using paired fold-level tests.

### 3.3 Five-fold cross-validation results

To evaluate the stability of the complete model across different data subsets, the model was trained independently on each stratified fold using RGB weights pretrained exclusively on the 13,952 non-paired RGB kernels. All 2,048 paired RGB–VNIR kernels used in the five-fold cross-validation had been excluded from the RGB pretraining set. As summarized in **Table 6**, the fold-wise Accuracy ranged from 98.53% to 99.02%, with a mean of 98.68% and a standard deviation of 0.22%. The narrow range of 0.49 percentage points indicates consistent performance across the five validation partitions. These results characterize the performance of the complete model under the present cross-validation protocol and should not be interpreted as independent test-set performance.

**Table 6:** Five-fold cross-validation results of the complete model.

Validation fold	Accuracy (%)
Fold 1	99.02
Fold 2	98.54
Fold 3	98.54
Fold 4	98.53
Fold 5	98.78
Mean ± SD	98.68 ± 0.22

For class-wise evaluation, the out-of-fold predictions from the five validation folds were pooled according to the original sample indices, ensuring that each of the 2,048 paired samples was included exactly once. Each class contained 256 samples, and the rows and columns of the resulting confusion matrix represent the true and predicted classes, respectively. As shown in **Figure 8**, the predictions are strongly concentrated along the main diagonal, demonstrating reliable discrimination across all eight quality categories. Of the 2,048 samples, 2,021 were correctly classified and 27 were misclassified. The most frequent errors occurred between insect-damaged and healthy-large kernels, and between broken-high and unhulled kernels, with five samples in each case. Minor confusion was also observed between broken-low and discolored-low, as well as between discolored-high and discolored-low. These errors suggest partial overlap in visual appearance or spectral response among specific category pairs rather than widespread ambiguity in the learned class boundaries. The underlying decision patterns are further examined using Grad-CAM++ and SHAP analyses.

**Figure 8:**
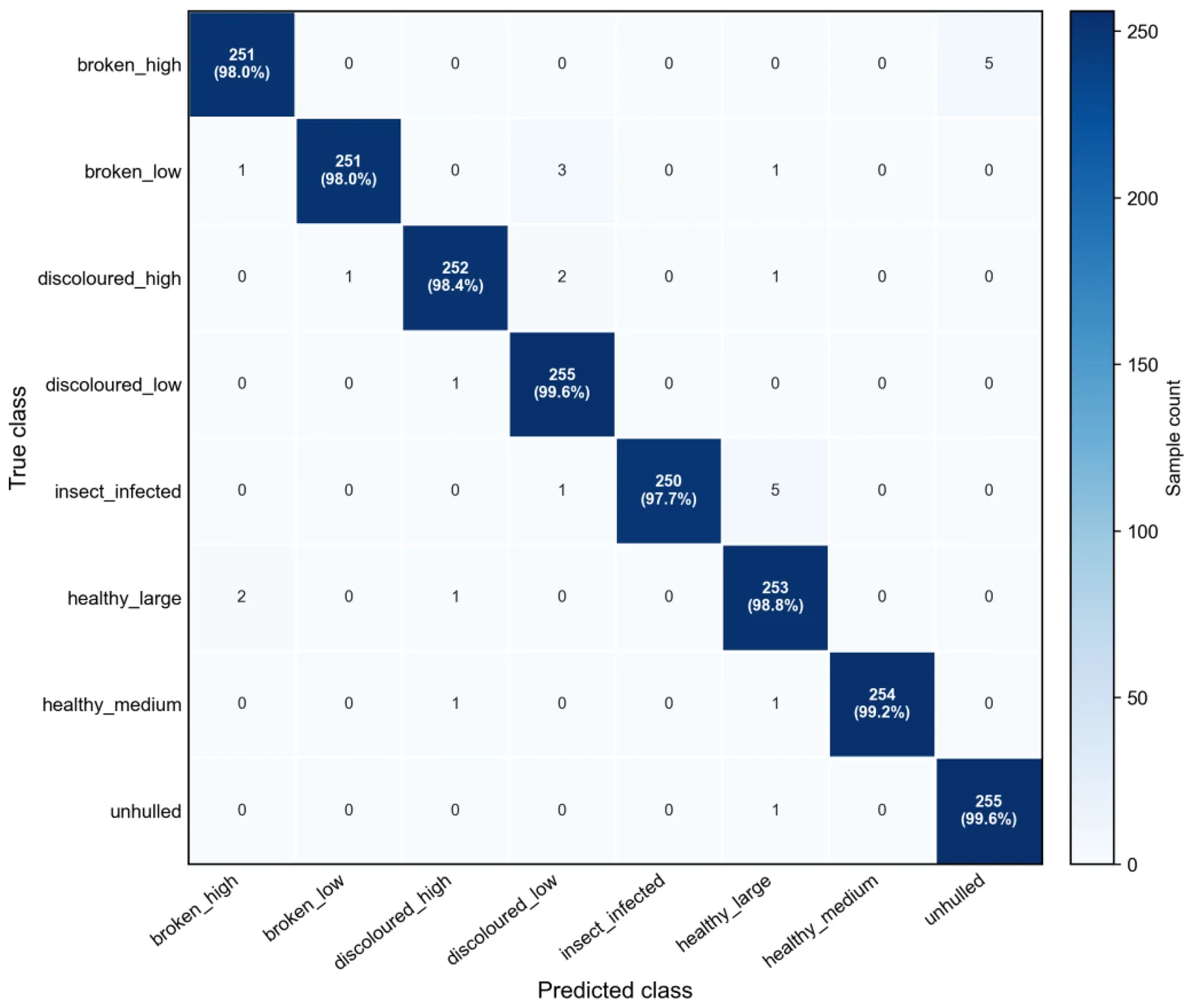
Aggregated confusion matrix of the complete model based on out-of-fold predictions from five-fold cross-validation.

As shown in **Figure 9**, both training and validation accuracies increased rapidly during the first 20 epochs and then improved more gradually. Although the validation curve exhibited slight fluctuations during the intermediate stage, it stabilized after approximately 50 epochs and remained close to the training curve. At the end of training, the two accuracies converged to approximately 99% and above 98%, respectively, indicating stable convergence without evident overfitting.

**Figure 9:**
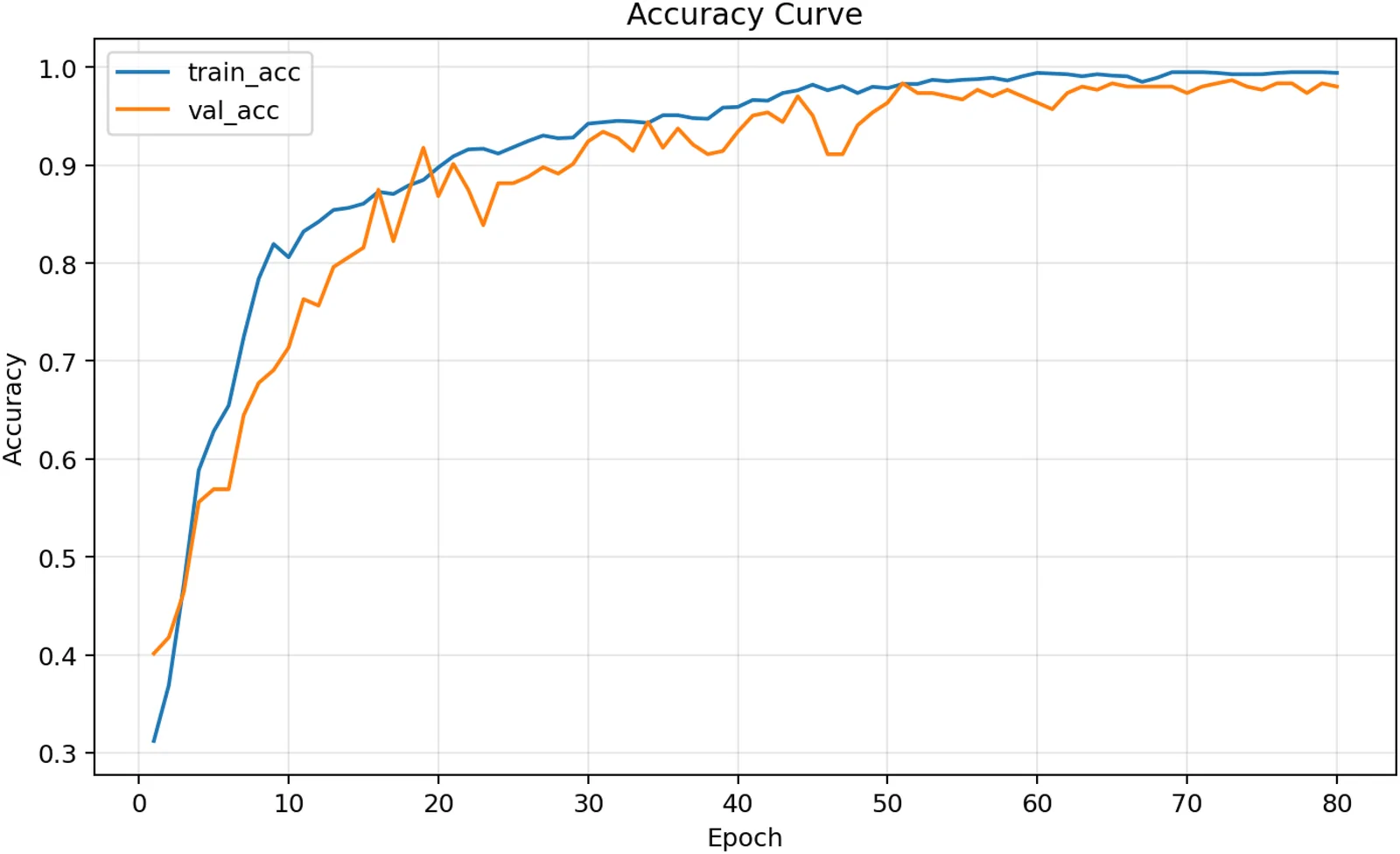
Training and validation accuracy curves of the complete model during training.

### 3.4 Spatial interpretability of the RGB branch

Gradient-weighted Class Activation Mapping++(Grad-CAM++ )was employed to visualize the spatial responses of the Coordinate Attention layer (Selvaraju et al., 2020). As shown in **Figure 10**, distinct activation patterns were observed across different quality categories. For broken-high kernels, the strongest responses were concentrated around the truncated end and its adjacent regions, corresponding closely to the visible damage. For broken-low kernels, the dominant activation appeared mainly in the middle-to-lower region, accompanied by weaker responses near the upper end. The responses for discolored-low and discolored-high kernels were distributed primarily over the central surface, suggesting that the RGB branch utilized color and texture cues when distinguishing discoloration severity. For insect-damaged kernels, strong activation was localized near visible abnormal regions, whereas healthy kernels exhibited broader responses over the central kernel body. The unhulled sample showed stronger activation in the upper-middle region. Overall, the high-response regions were generally aligned with the kernel body, damaged ends, and visible surface abnormalities, indicating that the RGB branch captured spatially relevant phenotypic cues. Nevertheless, some activation extended beyond the kernel boundaries, suggesting that the network may also utilize contextual information from the surrounding regions or imaging background. Such diffusion may additionally be related to the relatively low spatial resolution of the final convolutional feature maps.

**Figure 10:**
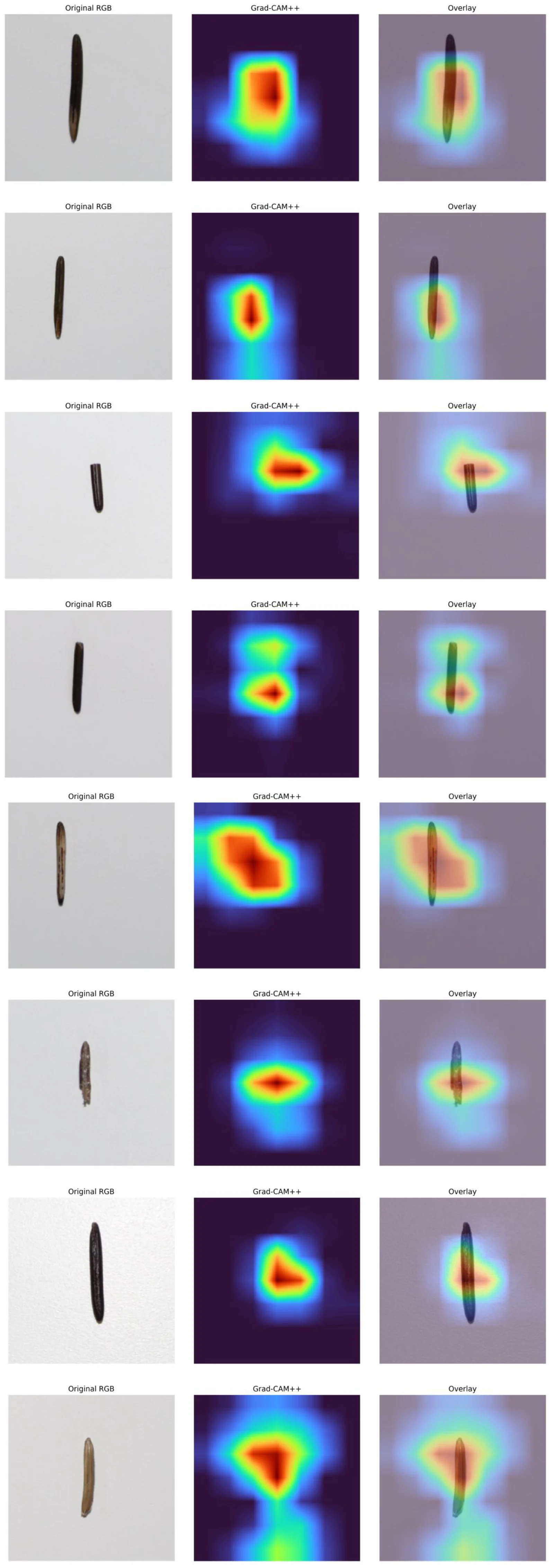
Grad-CAM++ visualizations of the coordinate attention responses for samples from different quality categories.

### 3.5 Wavelength interpretability of the VNIR branch

The VNIR branch was interpreted using SHAP GradientExplainer. During the analysis, the paired RGB input was held fixed, allowing the SHAP values to characterize the conditional contribution of each VNIR wavelength to the final multimodal prediction. The resulting global, class-specific, and sample-level explanations are presented on **Figure 11**.

**Figure 11:**
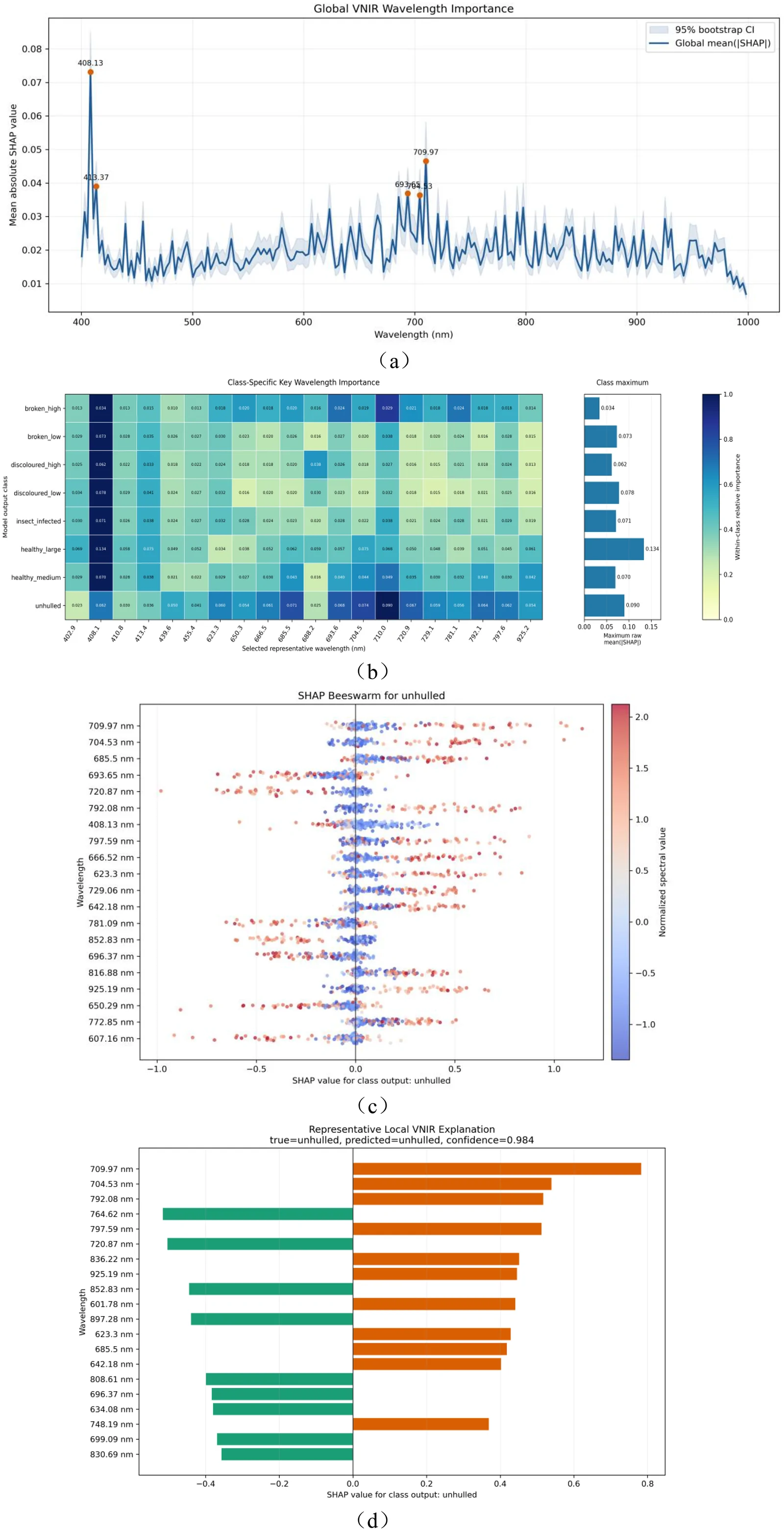
SHAP-based interpretation of the VNIR branch: **(a)** global wavelength importance with 95% bootstrap confidence intervals; **(b)** class-specific relative importance of representative wavelengths; **(c)** SHAP beeswarm plot for the unhulled category; and **(d)** local SHAP explanation of a representative correctly classified unhulled sample.

As shown in **Figure 11a**, the global mean absolute SHAP values were concentrated primarily around 408.13 nm and the 693.65–709.97 nm region, with 408.13, 709.97, 693.65, 704.53, and 413.37 nm exhibiting particularly high importance. The class-specific heatmap in **Figure 11b** further revealed that the relative contribution of individual wavelengths varied among categories. Although 408.13 nm contributed broadly across multiple classes, the unhulled category showed stronger dependence on wavelengths near 704.53 and 709.97 nm. These results indicate that the VNIR branch learned class-dependent combinations of spectral features rather than applying a uniform wavelength criterion to all categories. To examine the direction of wavelength contributions, a SHAP beeswarm plot was generated for the unhulled category, as shown in **Figure 11c**. Higher normalized spectral values at 704.53 and 709.97 nm were generally associated with positive SHAP values, whereas higher values at 693.65 and 720.87 nm were more frequently associated with negative contributions. At 408.13 nm, relatively lower spectral values tended to promote the unhulled prediction. This pattern suggests that the classification decision depended on the joint magnitude and direction of contributions from multiple wavelengths rather than on a single dominant spectral peak. The representative local explanation in **Figure 11d** showed that 709.97, 704.53, 792.08, and 797.59 nm contributed positively to the unhulled output, whereas 764.62, 720.87, 852.83, and 897.28 nm exerted negative effects. 704.53 and 709.97 nm were consistently highlighted in the global, class-specific, and local analyses, indicating agreement between the overall model behavior and the representative sample-level decision. By contrast, several wavelengths exhibited strong contributions only for the individual sample, reflecting the sample-dependent nature of spectral evidence. Overall, the SHAP results demonstrate that the VNIR branch integrates multiple wavelength regions through class- and sample-dependent contribution patterns. These explanations characterize the model’s decision behavior but do not establish direct causal relationships between the identified wavelengths and specific chemical constituents.

## 4 Discussion

The results demonstrate that RGB images and VNIR spectra provide complementary information for Canadian wild rice quality classification. Among the candidate backbones, the modified MobileNetV3-Small and 1D-GhostResNet-SE achieved the best performance for visual and spectral feature extraction, respectively. Under the same dual-branch configuration, CMCR-FH achieved numerically higher performance than the alternative fusion strategies. The complete model achieved strong validation performance while maintaining relatively low parameter counts and multiply–accumulate operations (MACs), indicating that its performance gains were primarily attributable to improved feature representation and cross-modal fusion rather than simply increased model complexity.

RGB images characterize spatial phenotypic features such as kernel shape, color, texture, breakage, and visible surface abnormalities, whereas VNIR spectra provide wavelength-dependent information related to reflectance, absorption, and scattering. The relative importance of these cues may vary across quality classes and individual samples because of differences in defect severity, hull coverage, and kernel condition. Consequently, direct concatenation or fixed-weight fusion may not adequately capture such heterogeneity. CMCR-FH addresses this limitation by dynamically adjusting the contribution of each modality according to their joint representation while retaining modality-specific information. This sample-dependent evidence integration provides a plausible explanation for why the dual-modality model consistently outperformed the RGB-only and VNIR-only models.

The Grad-CAM++ and SHAP analyses further supported the complementary roles of spatial and spectral information. Grad-CAM++ responses from the RGB branch were mainly concentrated on the kernel body, broken ends, and visibly abnormal regions, suggesting that the model relied on local phenotypic features relevant to quality classification. Some responses extended into the surrounding background, indicating that local contextual information may also have influenced the predictions. However, because pixel-level defect annotations were unavailable, these visualizations should be interpreted as qualitative evidence of spatial correspondence rather than precise defect localization or segmentation.

SHAP analysis identified influential VNIR responses around 408.13 nm and 693.65–709.97 nm. The response at 408.13 nm may be associated with differences in surface color, pigment status, and epidermal characteristics, whereas the red-to-near-infrared transition region may be influenced by pigment absorption, hull coverage, tissue structure, and surface scattering. These observations are broadly consistent with previous visible–near-infrared studies of rice quality and health status. 704.53 and 709.97 nm showed consistently strong contributions to the unhulled class, while the importance of other wavelengths varied across classes and samples. This pattern suggests that the VNIR branch relied on class- and sample-specific combinations of continuous spectral responses rather than on a single isolated wavelength.

Near-infrared responses may also contain information associated with O–H, C–H, and N–H molecular vibrations and have previously shown value in rice quality assessment and insect-damage detection. Nevertheless, the wavelengths identified by SHAP may be jointly affected by overlapping absorption, tissue scattering, and surface conditions. Their importance therefore cannot be attributed directly to a specific chemical constituent without targeted physicochemical validation. The remaining misclassifications were mainly observed between classes with similar phenotypic or spectral characteristics, indicating that the model established generally distinguishable class boundaries but still encountered difficulties when diagnostic cues overlapped, or defect severities were similar.

Several limitations should be acknowledged. The current dataset does not sufficiently cover variations across production years, geographical origins, batches, and imaging devices, and the model therefore requires independent external validation under changing acquisition conditions. Although the cross-validation results demonstrated consistent performance across the evaluated folds, they were obtained within the current development dataset. Further evaluation using an independent test set or nested cross-validation would provide a more rigorous assessment of model generalization and robustness on unseen data. In addition, the mutually exclusive labels do not fully represent compound defects, continuous severity levels, or multilabel conditions. The relationships between key wavelengths, tissue structures, and physicochemical properties also remain unverified, and the model still relies on relatively complete spectral information. Future studies should therefore expand the dataset across acquisition conditions, incorporate more realistic defect annotations, and combine spectral interpretation with targeted measurements of moisture, starch, protein, lipids, pigments, phenolic compounds, oxidation-related indicators, and microscopic structures. Key-wavelength selection, model calibration, domain adaptation, and conveyor-based online testing should also be investigated to reduce acquisition and computational costs while evaluating cross-device robustness, inference efficiency, predictive reliability, and long-term operational stability.

## 5 Conclusion

This study proposed a lightweight RGB–VNIR fusion method for the fine-grained classification of eight predefined postharvest categories of Canadian wild rice kernels. The proposed framework adaptively recalibrates the contributions of visual and spectral modalities, enabling complementary use of RGB phenotypic features and VNIR spectral information.

Using stratified five-fold cross-validation on 2,048 paired RGB–VNIR samples, the model achieved a mean accuracy of 98.68% ± 0.22% and a Macro-F1 score of 98.69% ± 0.22%, corresponding to numerical improvements of 4.93 and 4.97 percentage points over the baseline model, respectively. The ablation results showed that CMCR-FH produced the largest numerical performance gain among the evaluated components. Grad-CAM++ and SHAP analyses further indicated that the model jointly utilized visible phenotypic abnormalities and class-dependent spectral response patterns for category discrimination.

Overall, the proposed framework demonstrates the potential of sample-adaptive RGB–VNIR fusion for visual quality-category classification of Canadian wild rice kernels. Future work should incorporate independent physicochemical measurements and samples from different years, batches, and acquisition conditions to further evaluate the relationship between spectral responses and intrinsic quality attributes and to assess model generalization under practical conditions.

## Data Availability

The original contributions presented in the study are included in the article/supplementary material. Further inquiries can be directed to the corresponding author.
